# Supplementary material for: Intracellular delivery of protein drugs with an autonomously lysing bacterial system reduces tumor growth and metastases
Source: Nat Commun. 2021 Oct 21;12:6116. doi: 10.1038/s41467-021-26367-9 (PMC8531320; doi:10.1038/s41467-021-26367-9)
Supplement: Supplementary file 1 — Supplementary Information [file 41467_2021_26367_MOESM1_ESM.pdf]

Supplemental Information

Intracellular delivery of protein drugs with an autonomously lysing bacterial system reduces tumor growth and metastases

Vishnu Raman*[,1,2], Nele Van Dessel*[,1,2], Christopher L. Hall[1,2], Victoria E. Wetherby[2], Samantha A. Whitney[1], Emily L. Kolewe[1], Shoshana M.K. Bloom[1], Abhinav Sharma[1], Jeanne A. Hardy[3,4,5], Mathieu Bollen[6], Aleyde Van Eynde[6], Neil S. Forbes[1,2,4,5,†]

* Both authors contributed equally to this manuscript

[1]Department of Chemical Engineering
University of Massachusetts, Amherst
Amherst, MA, USA

[2]Ernest Pharmaceuticals, LLC
Hadley, MA, USA

[3]Department of Chemistry
University of Massachusetts, Amherst
Amherst, MA, USA

[4]Molecular and Cell Biology Program
University of Massachusetts, Amherst
Amherst, MA, USA

[5]Institute for Applied Life Science
University of Massachusetts, Amherst
Amherst, MA, USA

[6]Laboratory of Biosignaling & Therapeutics
Department of Cellular and Molecular Medicine
KU Leuven
Leuven, Belgium

[†]Corresponding author

Correspondence to:
Professor Neil S Forbes
Department of Chemical Engineering
University of Massachusetts, Amherst
159 Goessmann Laboratory
686 North Pleasant St.
Amherst, Massachusetts 01003, USA
Email: forbes@umass.edu
Tel.: +1 413 577 0132

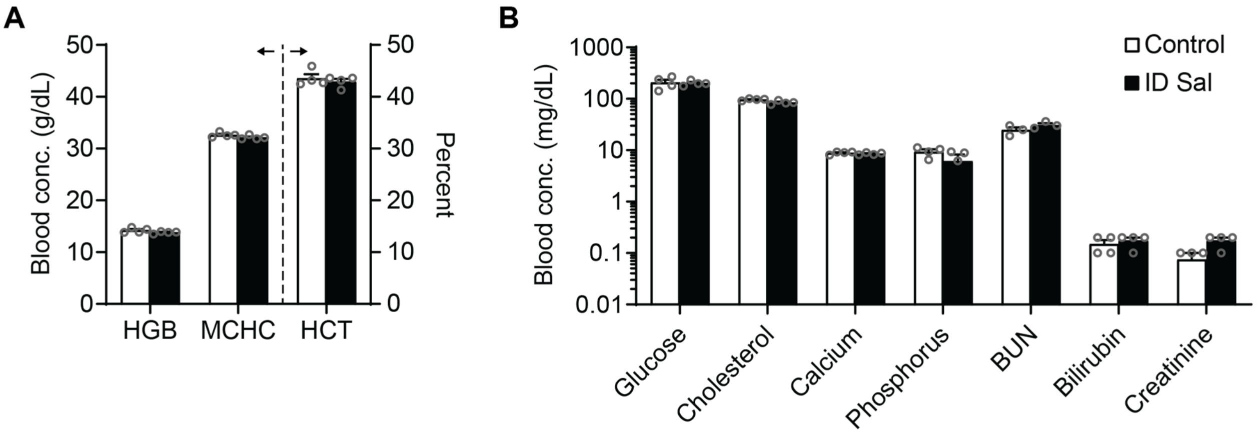

Supplementary Figure 1. Additional measures of blood or liver toxicity. A,B) Tumor-free BALB/c mice were injected with $1{\times}10^7$ ID Salmonella, and control mice were injected with saline (n = 4). After 14 days, whole blood was isolated by percutaneous cardiac puncture. Comprehensive hematology (***A***) and chemistry profiling (***B***) was conducted by *Idexx Laboratories* (Grafton, MA). No differences were detected for any measurement of blood or liver toxicity. Abbreviations are BUN, blood urea nitrogen; HCT, hematocrit; HGB, hemoglobin; and MCHC, mean corpuscular hemoglobin concentration. Data are shown as means ± SEM.

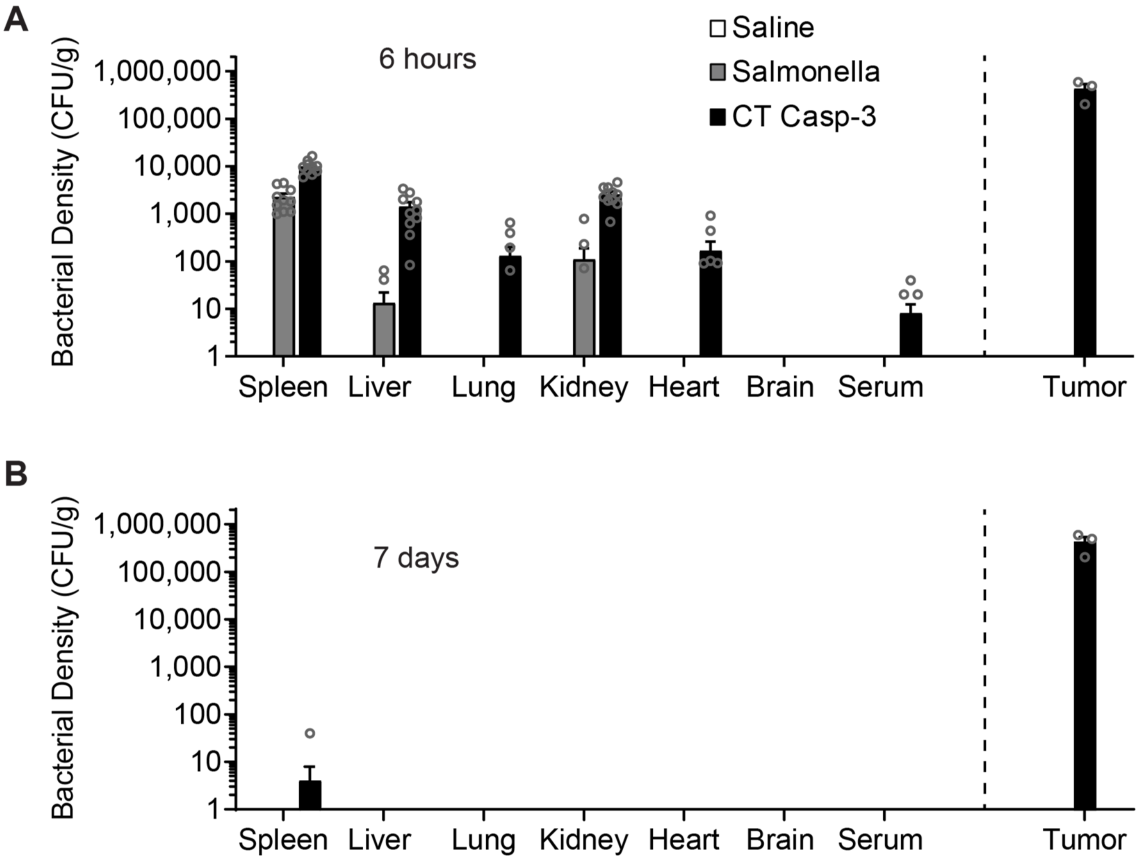

Supplementary Figure 2. Biodistribution of *CT Casp-3* Salmonella at 6 hours and 7 days. Tumor-free male C57BL/J mice were intravenously injected at 22 weeks of age with either saline (control), $1x10^7$ CFU/mouse of ID Salmonella, or $1x10^7$ CFU/mouse of *CT Casp-3* Salmonella with (*n* = 10 for both time points). **A)** After 6 h, tissues were excised and weighed: spleen, liver, lung, kidney, heart, brain and serum. Organs were minced and cultured on agar plates. The bacterial density in the tumors of tumor-bearing mice (from Figure 5b) at 72 h is provided as a reference. No bacteria were detected in saline controls. **B)** After 7 days, bacterial density was determined from an additional set of identically treated mice. No bacteria were detected in most of the organs. The bacterial density in the spleens of mice treated with *CT Casp-3* Salmonella was close to the detection limit. The presence of bacteria in healthy organs is common six hours after intravenous injection (as seen in *a*). At this time, bacteria are still present in the blood in these organs. Because the bacteria do not interact with the tissues, no adverse effects were seen (as shown in Figures 5c-e, S1 and S4). With time, the immune system clears the bacteria from the blood and organs, typically within 24 h, as can be seen by the lack of bacteria in most organs after 7 days (*b*). Data are shown as means ± SEM.

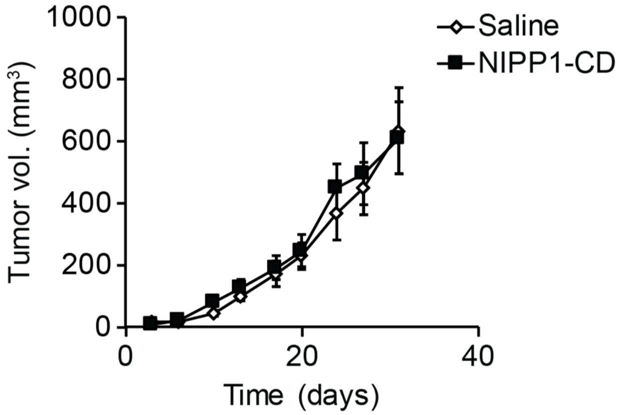

Supplementary Figure 3. Efficacy of ID Salmonella delivery of NIPP1-CD. *NIPP1-CD* Salmonella (1×10^7 CFU/mouse) were administered to BALB/c mice with subcutaneous 4T1 tumors by intravenous injection. No difference in tumor volume was observed between NIPP1-CD (n = 5) and saline controls (n = 4). Data are shown as means ± SEM.

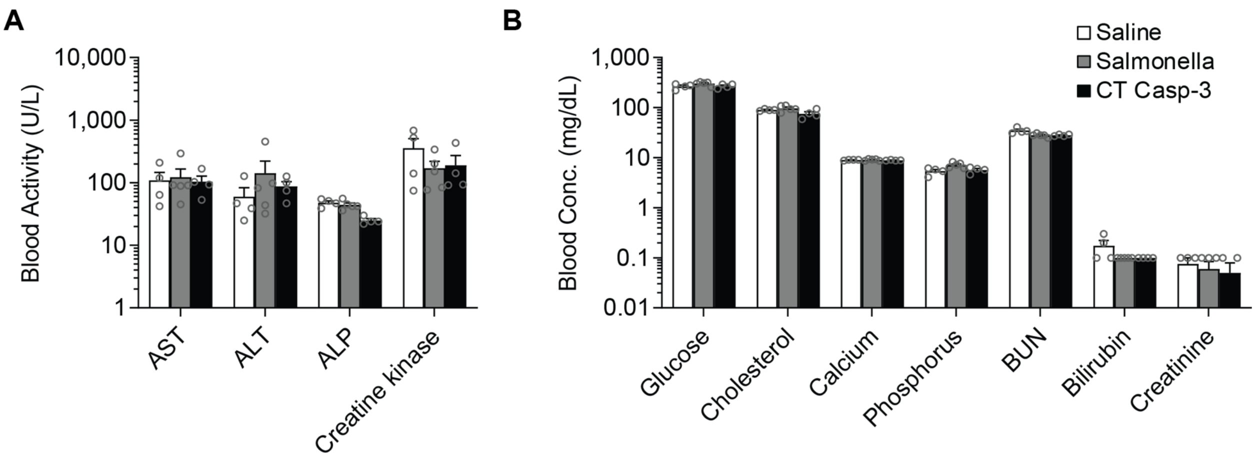

Supplementary Figure 4. Toxicity of ID Salmonella with CT Casp-3. A) Chemistry profiling of the blood drawn from tumor-free male C57BL/J mice (*n* = 4). Mice were intravenously injected at 22 weeks of age with either saline (control), $1x10^7$ CFU/mouse of ID Salmonella, or $1x10^7$ CFU/mouse of ID Salmonella with CT Casp-3. Sera were collected seven days after injection and chemistry profiling was conducted by *IDEXX Laboratories* (Grafton, MA). No differences were detected for any measurement of liver toxicity. Markers of liver damage are ALP, alkaline phosphatase; ALT, alanine transaminase; and AST, aspartate transaminase. **B)** Additional measures of liver toxicity of the blood from the mice in (A). No differences were detected for any measurement. BUN is blood urea nitrogen. Data are shown as means ± SEM.

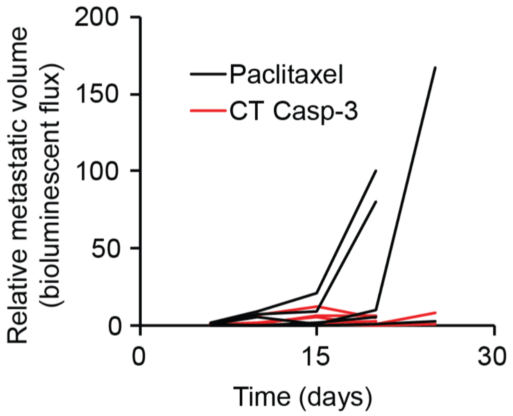

Supplementary Figure 5. ***CT Casp-3*** **Salmonella prevented growth of metastases in individual mice.** Lung metastases were formed in BALB/c mice by intravenous injection of $5×10^4$ luciferase-expressing 4T1 cells (see Figure 7G). Mice were treated with either $1×10^7$ CFU of *CT Casp-3* Salmonella or 10 mg/kg paclitaxel (*n* = 6). Metastatic volume was measured with bioluminescence imaging after IP injection of 100 µl of 30 mg/ml D-luciferin, and is reported relative to initial volume. Metastases grew exponentially in three mice treated with paclitaxel, but did not grow in any mice treated with CT Casp-3.

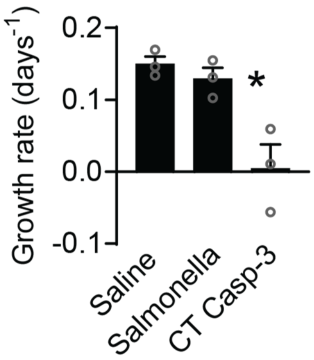

Supplementary Figure 6. Tumor growth rate. In C57L/J mice with subcutaneous Hepa 1-6 liver tumors (Figure 7I-J), treatment with *CT Casp-3* Salmonella significantly reduced tumor growth rate compared to both saline and bacterial (ID Salmonella) controls (*, P = 0.027; *n* = 3). Data are shown as means ± SEM. The statistical comparison is a two-tailed, unpaired Student's t test. The asterisk (*) indicates significance of P < 0.05.

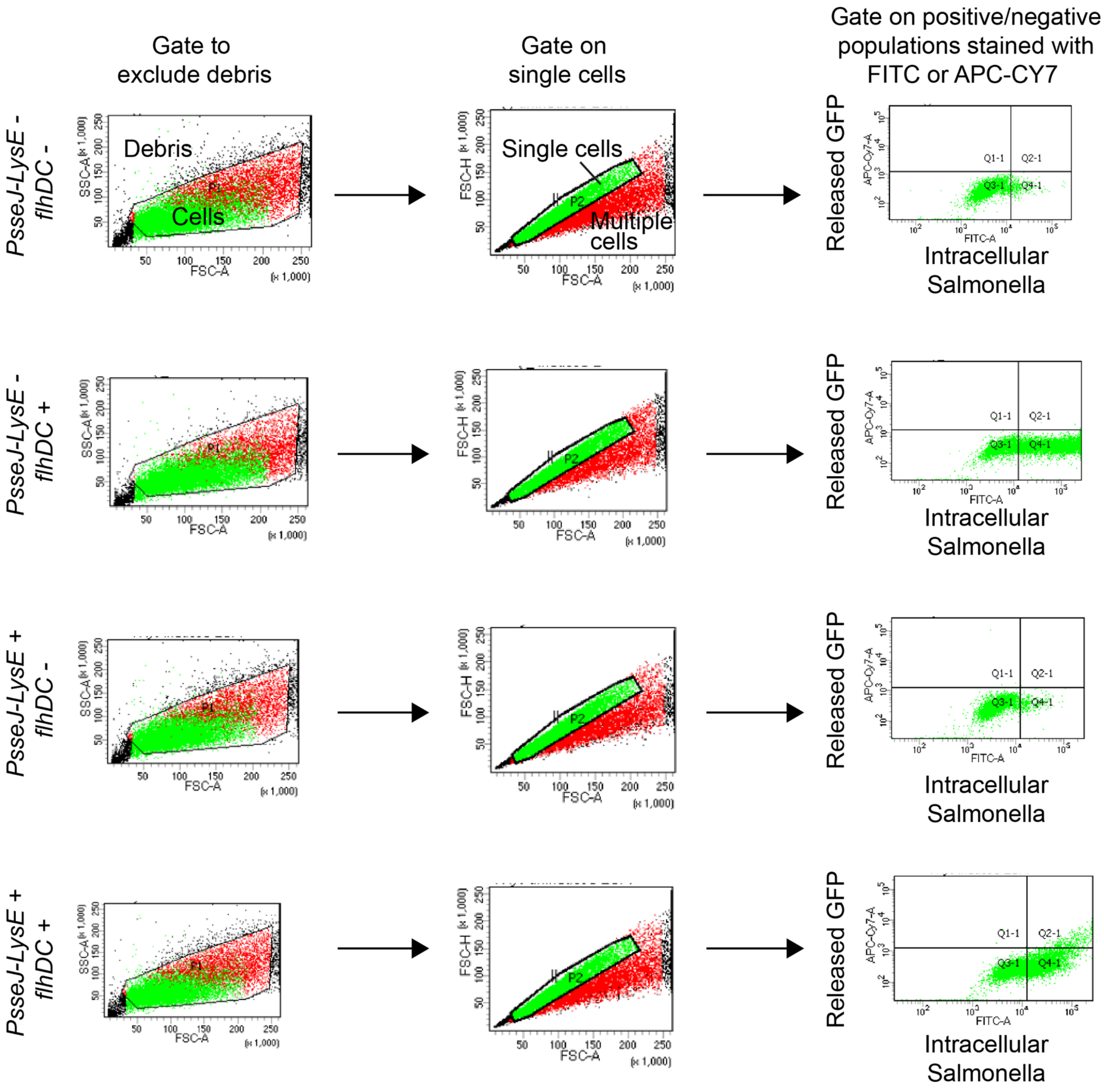

Supplementary Figure 7. Flow cytometry gating strategy. IDf+ Salmonella were administered to 4T1 cancer cells (see Figure 4B). Four different conditions were investigated: IDf+ Salmonella without *Pssej-LysE* and without arabinose induction of *PBAD-flhDC* (*Pssej-LysE* -, *flhDC* -); without *Pssej-LysE* and with induction of *PBAD-flhDC* with 20 mM arabinose (*Pssej-LysE* -, *flhDC* +); with *Pssej-LysE* and without induction (*Pssej-LysE* +, *flhDC* -); with *Pssej-LysE* and with induction (*Pssej-LysE* +, *flhDC* +). After invasion and delivery, the 4T1 cells were harvested, fixed and stained for *Salmonella* (FITC) and myc-tagged GFP (Dylight 755; APC-CY7). The gating strategy for each condition is shown. Cells were first gated to exclude debris and then gated on single cells. FITC and APC-CY7 gates were used to quantify the percentage of cells with intracellular Salmonella and released GFP-myc, respectively. The four rightmost plots are the images in Figure 4B of the manuscript.

Supplementary Table 1. Bacterial strains

Strain	Background / Knockouts	Plasmid	Genetic functions	Description
Parental (Par)	*ΔmsbB*, *ΔpurI*, *Δxyl*, *Δasd*	-	-	Non-pathogenic therapeutic Salmonella; deletion of *asd* enables balanced lethal system to maintain plasmids in vivo
Δ*flhD* Sal	Δ*flhD Par*	-	-	Parental Salmonella with *flhD* deletion; non-motile
Intracellular reporting	*Par*	P1	*PsseJ-GFP*	Intracellularly inducible GFP
flhDC Sal	Δ*flhD Par*	P2	*PBAD-flhDC*<br>*Plac-GFP*	Re-expresses *flhDC* after induction with arabinose<br>Constitutively expresses GFP
flhDC reporting	Δ*flhD Par*	P4	*PBAD-flhDC*<br>*PsseJ-GFP*	Re-expresses *flhDC* after induction with arabinose<br>Intracellularly inducible GFP
PsifA	*Par*	P5	*PsifA-GFP*	Expresses GFP after activation of *PsifA* promoter
PBAD-LysE	*Par*	P6	*PBAD-LysE*	Bacteria lyse after activation with arabinose
ID Sal	*Par*	P7	*PsseJ-LysE*<br>*Plac-GFP*	Bacteria lyse after activation of *PsseJ* promoter<br>Constitutively expresses GFP
Δ*sifA* ID Sal	Δ*sifA Par*	P7	*PsseJ-LysE*<br>*Plac-GFP*	Predominantly accumulates in the cytoplasm of cells<br>Lyses after invasion<br>Constitutively expresses GFP
Δ*sseJ* ID Sal	Δ*sseJ Par*	P7	*PsseJ-LysE*<br>*Plac-GFP*	Predominantly accumulate in SCVs<br>Lyses after invasion<br>Constitutively expresses GFP
IDf+ Sal	Δ*flhD Par*	P8	*Plac-GFP*<br>*PBAD-flhDC*<br>*PsseJ-LysE*	Re-expresses *flhDC* after induction with arabinose<br>Lyses after invasion<br>Constitutively expresses GFP
ID Sal-luc	*Par*	P9	*PsseJ-LysE*<br>*Plac-GFP*<br>*Plac-luc*	Bacteria lyse after activation of *PsseJ* promoter<br>Constitutively expresses GFP and luciferase
NB	*Par*	P10	*PsseJ-LysE*<br>*PBAD-nano*<br>*Plac-GFP*	Lyses after invasion<br>Controllably expresses nanobody against β-actin

				Constitutively expresses GFP
NIPP1-CD Sal	*Par*	P11	*PsseJ-LysE*<br>*Plac-NIPP1*<br>*Plac-GFP*	Lyses after invasion<br>Constitutively expresses NIPP1-CD and GFP
CT Casp-3 Sal	*Par*	P12	*PsseJ-LysE*<br>*PBAD-Casp3*<br>*Plac-GFP*	Lyses after invasion<br>Controllably expresses CT Casp-3<br>Constitutively expresses GFP

Supplementary Table 2. Plasmids

No.	Name	Origin	Gene Main-tenance	Gene Circuits	Purpose
P1	Intracellular reporting	ColE1	Chlor[a] ASD[b]	*PsseJ-GFP*	Expresses GFP after cell invasion
P2	*flhDC* re-expressing	ColE1	Amp ASD	*PBAD-flhDC* *Plac-GFP-myc*	Re-expresses *flhDC*; Constitutively expresses GFP
P3	*PBAD-flhDC*	ColE1	Amp[c]	*PBAD-flhDC*	Used in construction of P2
P4	*flhDC* reporting	ColE1	Amp	*PBAD-flhDC* *PsseJ-GFP-myc*	Measures invasion after *flhDC* re-expression
P5	*PsifA* reporter plasmid	ColE1	Chlor ASD	*PsifA-GFP*	Expresses GFP after activation of *PsifA* promoter
P6	Inducible lysis	ColE1	Chlor ASD	*PBAD-LysE*	Lyses after activation with arabinose
P7	Intracellular lysis	ColE1	Chlor ASD	*PsseJ-LysE* *Plac-GFP-myc*	Bacteria lyse after invasion; Constitutively expresses GFP
P8	Intracellular lysis and induced invasion	ColE1	AMP ASD	*PsseJ-LysE* *PBAD-flhDC* *Plac-GFP-myc*	Lyses after invasion; Re-expresses *flhDC*; Constitutively expresses GFP
P9	Luciferase	ColE1	Chlor ASD	*PsseJ-LysE* *Plac-GFP-myc* *Plac-luc*	Bacteria lyse after invasion; Constitutively expresses GFP and luciferase
P10	Nanobody	ColE1	AMP ASD	*PsseJ-LysE* *PBAD-nano-flag*	Lyses after invasion; Expresses flag-tagged nanobody against β-actin
P11	NIPP1-CD	ColE1	Chlor ASD	*PsseJ-LysE Plac-NIPP1*	Lyses after invasion; Expresses NIPP1-CD
P12	CT Casp-3	ColE1	AMP ASD	*Plac-GFP* *PsseJ-LysE* *PBAD-Casp-3*	Lyses after invasion; Constitutively expresses GFP; Expresses CT Casp-3

[a]Chloramphenicol

[b]ASD (aspartate-semialdehyde dehydrogenase) is an essential enzyme for lysine synthesis and is necessary for the synthesis of peptidoglycan[4]. It is the key gene in the balanced lethal system developed by Nakayama et al.[5] to maintain genes in Salmonella after injection in vivo.

[c]Ampicillin

Supplementary Table 3. Primers used for gene deletions

Name	Primer sequence	Gene	Template
vr121	FOFZCACGGGGTGCGGCTACGTCGCACAAAAATAAAGTTGGTTATTCTGGGTCTTGAGCGATTGTGTAGGC	*flhD* forward	pkd4
vr309	EOEFATCCTGAGTCAAACGGGTGATCGTCTGATGATCGTCAAACCGGAAAAATTAGCCATGGTCCATATGAATATC	*flhD* reverse	pkd4
vr266	FZEFAAAATGTTGGTTTTATCGGCTGGCGCGGAATGGTCGGCTCTGTTCTGTCTTGAGCGATTGTGTAGGC	*asd* forward	pkd3
vr268	OZFOGCCAACTGGCGCAGCATTCGACGCAGCGGCTCGGCGGCGCCCCATAAATTAGCCATGGTCCATATGAATATC	*asd* reverse	pkd3
vr432	FZEOCATTGAGTGTTGGACAGGGTTATTTCACATCATCTATCAGTTCTGAGTCTTGAGCGATTGTGTAGGC	*sseJ* forward	pkd4
vr433	ZZFZTCAGTGGAATAATGATGAGCTATAAAACTTTCTAACATTATGGCAAAATTAGCCATGGTCCATATGAATATC	*sseJ* reverse	pkd4
vr434	FZEOCGATTACTATAGGGAATGGTTTTTTAAAAAGTGAAATCCTTACCAAGTCTTGAGCGATTGTGTAGGC	*sifA* forward	pkd4
vr435	ZZFZAAAAAACAACATAAACAGCCGCTTTGTTGTTCTGAGCGAACGTGTAAATTAGCCATGGTCCATATGAATATC	*sifA* reverse	pkd4

Supplementary Table 4. Primers used for plasmid construction

Name	Sequence	Description
nd1	AAAAAAACATGTGTGGAATTGTGAGCGGATAAC	*Plac-GFP* forward
nd2	AAAAAAGACGTCTTATTTGTATAGTTCATCCATGCC	*Plac-GFP* reverse
nd3	AAAAAAACATGTCACATAAAACACTAGCACTTT	*PsseJ* forward
nd4	AAAAAATCTAGACCTCCTTACTTTATTAAACAC	*PsseJ* reverse
nd5	AAAAAAACATGTTATAAGCGATTAATTGCGCAA	*PsifA* forward
nd6	AAAAAATCTAGATAATCTCACTTATACTGGAGT	*PsifA* reverse
nd7	AAAAAACCATGGTTTAAGAAGGAGATATACATATGG	*LysE* forward (into PBAD)
nd8	AAAAAAGGTACCTCACTCCTTCCGCACGTAATT	*LysE* reverse (into PBAD)
nd9	AAAAAATCTAGATTTAAGAAGGAGATATACATATGG	*LysE* forward
nd10	AAAAAAGACGTCTCACTCCTTCCGCACGTAATT	*LysE* reverse
nd11	AAAAAA GAGCTC GACTGGAAAGCGGGCAGTGA	*Plac-GFP* forward
nd12	AAAAAA GAGCTC AAGCTTGCATGCCTGCAGGAG	*Plac-GFP* reverse
nd13	TTTAAGAAGGAGATATACATATGGGTGGAGAGGATGATG	*NIPP1* forward
nd14	CTTGCATGCCTGCAGGAGATTTACAGATCCTCTTCTGAGAT GAGTTTTTGTTCGTTCCGAAAGCGACCAAC	*NIPP1* forward
nd15	ATGTATATCTCCTTCTTAAATCTAGAGGTC	*Plac-GFP* backbone forward
nd16	GAACAAAAACTCATCTCAGAAGAGGATCTGTAAATCTCCTG CAGGCATGCA	*Plac-GFP* backbone reverse
nd17	AAAAAA GAGCTC GTTAGCAATTTAACTGTGATAAAC	*Plac-NIPP1-CD* reverse
ch1	TCAATCTCCTGCAGGCATGCTTTACACTTTATGCTTCCGGC TCGTATAATAAAAAAAAAAAAGGAGGAAAAAAAATGGAAGA TGCCAAAAACATTAAGAA	Luciferase forward
ch2	GGGGCGTAATTTGATATCAAGCTTTACACGGCGATCTTGCC G	Luciferase reverse
ch3	AGCTTGATATCAAATTACGCCCC	P6 backbone forward
ch4	GCATGCCTGCAGGAGATTGA	P6 backbone reverse
vr46	AAAAAACCATGGGTTAATAAAAGGAGGAATATATATGCATA CATCCGAGTTGCTAAAACA	*flhDC* forward
vr47	AAAAAACTCGAGAAAAATTAAACAGCCTGTTCGATCTGTTCA T	*flhDC* reverse
vr394	CCGCATAGTTAAGCCAGTATACATTTACACTTTATGCTTCCG GCTCGTATAATAAAAAAAAAAGGAGGAAAAAAAATGAGTAA AGGAGAAGAACTTTTCA	*Plac-GFP* backbone forward
vr395	TCACGTAGCGATAGCGGAGTTACAGATCCTCTTCTGAGATG AGTTTTTGTTCTTTGTATAGTTCATCCATGCCAT	*Plac-GFP* backbone reverse
vr385	CTCCGCTATCGCTACGTGA	P3 backbone forward
vr386	TGTATACTGGCTTAACTATGCGG	P3 backbone reverse

vr424	GCTTGTCTGCTCCCGGCATCGTACGTTTTCGTTCCATTGG	*asd* forward
vr425	AGACGGTCACAGCTTGTCTGTATCTGCGTTTACTCCTGTAT TAC	*asd* reverse
vr426	ACAGACAAGCTGTGACCGTCT	backbone forward
vr427	ATGCCGGGAGCAGACAAGC	backbone reverse
vr269	CGCAGCGAGTCAGTGAGCACATGTCACATAAAACACTAGC ACT	*Pssej-GFP-myc* forward
vr270	CGCACAGATGCGTAAGGAGAATTACAGATCCTCTTCTGAGA TGAGTTTTTGTTCTTTGTATAGTTCATCCATGCCATG	*Pssej-GFP-myc* reverse
vr271	GCTCACTGACTCGCTGCG	P3 backbone reverse
vr272	TTCTCCTTACGCATCTGTGCG	P3 backbone forward
vr398	ATCTGTGCGGTATTTCACACCACATGTCACATAAAACACTA GCACT	*Pssej-LysE* forward
vr399	TACTGAGAGTGCACCATATGCTCACTCCTTCCGCACGTAAT TT	*Pssej-LysE* reverse
vr396	GCATATGGTGCACTCTCAGTA	P2 backbone forward
vr397	GGTGTGAAATACCGCACAGAT	P2 backbone reverse
vr466	GGGCTAACAGGAGGAATTAACCATGGCTCAGGTGCAGCTG G	Actin nanobody forward
vr467	TACCAGCTGCAGATCTCGAGTTACTTGTCGTCATCGTCTTT GTAGTCCATGCTTCTTGAGGAGACGGTGA	Actin nanobody reverse
vr448	CTCGAGATCTGCAGCTGGTA	P8 backbone forward
vr449	GGTTAATTCCTCCTGTTAGCCC	P8 backbone reverse
vr450	GGGCTAACAGGAGGAATTAACCATGGACTACAAAGACGAT GACGACAAGATGGAGAACACTGAAAACTCAGTG	N-term FLAG-Casp forward
vr451	TACCAGCTGCAGATCTCGAGTTACAGATCCTCTTCTGAGAT GAGTTTTTGTTCGTGATAAAAATAGAGTTCTTTTGTGAG	C-term myc-Casp3 reverse

Supplementary References

1 Mosberg, J. A., Lajoie, M. J. & Church, G. M. Lambda red recombineering in Escherichia coli occurs through a fully single-stranded intermediate. *Genetics* **186**, 791-799, doi:10.1534/genetics.110.120782 (2010).

2 Swofford, C. A., Van Dessel, N. & Forbes, N. S. Quorum-sensing Salmonella selectively trigger protein expression within tumors. *Proceedings of the National Academy of Sciences of the United States of America* **112**, 3457-3462, doi:10.1073/pnas.1414558112 (2015).

3 Vaidya, S., Velazquez-Delgado, E. M., Abbruzzese, G. & Hardy, J. A. Substrate-induced conformational changes occur in all cleaved forms of caspase-6. *J Mol Biol* **406**, 75-91, doi:10.1016/j.jmb.2010.11.031 (2011).

4 Yan, Y. *et al.* Asd-based balanced-lethal system in attenuated Edwardsiella tarda to express a heterologous antigen for a multivalent bacterial vaccine. *Fish & shellfish immunology* **34**, 1188-1194, doi:10.1016/j.fsi.2013.01.027 (2013).

5 Nakayama, K., Kelly, S. M. & Curtiss, R. Construction of an ASD+ expression-cloning vector - stable maintenance and high-level expression of cloned genes in a Salmonella vaccine strain. *Bio-Technol* **6**, 693-697, doi:10.1038/nbt0688-693 (1988).
