# Supplementary material for: Intracellular delivery of protein drugs with an autonomously lysing bacterial system reduces tumor growth and metastases
Source: Nat Commun. 2021 Oct 21;12:6116. doi: 10.1038/s41467-021-26367-9 (PMC8531320; doi:10.1038/s41467-021-26367-9)
Supplement: Supplementary file 2 — Reporting Summary [file 41467_2021_26367_MOESM2_ESM.pdf]

nature portfolio

Corresponding author(s): Neil Forbes

Last updated by author(s): Sep 27, 2021

Reporting Summary

Nature Portfolio wishes to improve the reproducibility of the work that we publish. This form provides structure for consistency and transparency in reporting. For further information on Nature Portfolio policies, see our Editorial Policies and the Editorial Policy Checklist.

Statistics

For all statistical analyses, confirm that the following items are present in the figure legend, table legend, main text, or Methods section.

n/a	Confirmed	
☐	☒	The exact sample size (*n*) for each experimental group/condition, given as a discrete number and unit of measurement
☐	☒	A statement on whether measurements were taken from distinct samples or whether the same sample was measured repeatedly
☐	☒	The statistical test(s) used AND whether they are one- or two-sided<br>*Only common tests should be described solely by name; describe more complex techniques in the Methods section.*
☐	☒	A description of all covariates tested
☐	☒	A description of any assumptions or corrections, such as tests of normality and adjustment for multiple comparisons
☐	☒	A full description of the statistical parameters including central tendency (e.g. means) or other basic estimates (e.g. regression coefficient) AND variation (e.g. standard deviation) or associated estimates of uncertainty (e.g. confidence intervals)
☐	☒	For null hypothesis testing, the test statistic (e.g. *F*, *t*, *r*) with confidence intervals, effect sizes, degrees of freedom and *P* value noted<br>*Give P values as exact values whenever suitable.*
☒	☐	For Bayesian analysis, information on the choice of priors and Markov chain Monte Carlo settings
☒	☐	For hierarchical and complex designs, identification of the appropriate level for tests and full reporting of outcomes
☒	☐	Estimates of effect sizes (e.g. Cohen's *d*, Pearson's *r*), indicating how they were calculated

Our web collection on statistics for biologists contains articles on many of the points above.

Software and code

Policy information about availability of computer code

Data collection	Excel (Microsoft Office Professional Plus 2016), Matlab (R2020a), BD FACSDiva (8.0)
Data analysis	Excel (Microsoft Office Professional Plus 2016), Matlab (R2020a), GraphPad Prism (9.2.0), BD FACSDiva (8.0)

For manuscripts utilizing custom algorithms or software that are central to the research but not yet described in published literature, software must be made available to editors and reviewers. We strongly encourage code deposition in a community repository (e.g. GitHub). See the Nature Portfolio guidelines for submitting code & software for further information.

Data

Policy information about availability of data

All manuscripts must include a data availability statement. This statement should provide the following information, where applicable:

- Accession codes, unique identifiers, or web links for publicly available datasets
- A description of any restrictions on data availability
- For clinical datasets or third party data, please ensure that the statement adheres to our policy

All data generated in this study are available within the Article, Supplementary Information or Source Data file. Source data generated in this study have been also deposited in the figshare database with digital identifier https://doi.org/10.6084/m9.figshare.16439073.

Field-specific reporting

Please select the one below that is the best fit for your research. If you are not sure, read the appropriate sections before making your selection.

☒ Life sciences ☐ Behavioural & social sciences ☐ Ecological, evolutionary & environmental sciences

For a reference copy of the document with all sections, see nature.com/documents/nr-reporting-summary-flat.pdf

Life sciences study design

All studies must disclose on these points even when the disclosure is negative.

Sample size	For tumor efficacy experiments in mice, sample size was based on similar experiments previously conducted by our group. From these experiments, it was determined that a three mouse sample would enable detection of volume differences (164.6, 191.4, 230.1, and 263.1 mm^3) at powers of 70, 80, 90, and 95%. This sample size yields adequate power to detect differences greater than 191.4 mm^3 that correspond to meaningful improvement in outcome compared to control mice (569.3 mm^3). For in vitro experiments, sample size was based on previous studies with similar experiments. For all experiments, at least three independent samples were analyzed, which provided sufficient power to perform unpaired Student's t tests or ANOVA analysis.
Data exclusions	For tumor efficacy experiments in mice, exclusion criteria were pre-established and set as part of the animal protocol approved by the UMass Institutional Animal Care and Use Committee (IACUC). All dead mice and mice with tumors exceeding 1000 m^3 were removed from the studies. These criteria were applied to mice in Figures 7 (e) and (h)-(j).
Replication	Many of the experiments were repeated multiple times with the independent biological samples using similar experimental conditions as mentioned in the figure legends. These include the delivery of GFP to tumors in mice; the non-toxicity of ID Salmonella in tumor-free mice; and the efficacy of CT Casp-3 Salmonella in multiple tumor models. All attempts at replication were successful.
Randomization	For all in vitro experiments, samples were randomly allocated into experimental groups. For animal experiments, mice were randomly allocated into treatment groups.
Blinding	For all studies, investigators were blinded to group allocation during data collection and analysis. For mechanistic animal and in vitro studies, group allocation was blinded by uniformly collecting all samples and performing all analyses in parallel. For efficacy studies in mice, blinding was achieved by having one investigator allocate mice into groups with blinded identifiers and having a second investigator measure tumor size.

Reporting for specific materials, systems and methods

We require information from authors about some types of materials, experimental systems and methods used in many studies. Here, indicate whether each material, system or method listed is relevant to your study. If you are not sure if a list item applies to your research, read the appropriate section before selecting a response.

Materials & experimental systems

n/a	Involved in the study	
☐	☒	Antibodies
☐	☒	Eukaryotic cell lines
☒	☐	Palaeontology and archaeology
☐	☒	Animals and other organisms
☒	☐	Human research participants
☒	☐	Clinical data
☒	☐	Dual use research of concern

Methods

n/a	Involved in the study	
☒	☐	ChIP-seq
☐	☒	Flow cytometry
☒	☐	MRI-based neuroimaging

Antibodies

Antibodies used

All antibodies were standard and commercially available. For each, the supplier and catalog number are as follows:

1. Rabbit polyclonal anti-Salmonella (Abcam, ab35156, lot GR3244696-2)
2. Rabbit polyclonal anti-Salmonella (Abcam, ab69253, lot GR3186947)
3. Rat monoclonal anti-myc (Chromotek, 9e1-100, lot 70117003AB)
4. Rabbit polyclonal anti-LAMP1 (Abcam, ab24170, lot GR3175891)
5. Rat monoclonal anti-GFP (Chromotek, 3h9-100, lot 60706001AB)
6. Rabbit polyclonal anti-GFP (Abcam, ab6556)
7. Goat polyclonal anti-GPF (Abcam, ab6673, lot GR241388-1)
8. Mouse monoclonal anti-β-actin (GeneTex, GTX26276)
9. Mouse monoclonal anti-β-actin (Cell Signaling Technology, 8H10D10, lot 17)

Validation	The validation of all primary antibodies is provided on the website of the suppliers, as listed below.<br><br>1) Salmonella (Abcam, ab35156): Rabbit. ELISA. [https://www.abcam.com/salmonella-antibody-ab35156.html]<br>2) Salmonella (Abcam, ab69253): Rabbit. ICC, IF. [https://www.abcam.com/fitc-salmonella-antibody-ab69253.html]<br>3) myc (Chromotek, 9e1-100): Rat. WB, IF, ELISA, IP. [https://www.chromotek.com/products/detail/product-detail/myc-tag-antibody-9e1/]<br>4) LAMP1 (Abcam, ab24170): Rabbit. WB, IHC-P [https://www.abcam.com/lamp1-antibody-lysosome-marker-ab24170.html]<br>5) GFP (Chromotek, 3h9-100): Rat. WB. [https://www.chromotek.com/products/detail/product-detail/gfp-antibody-3h9/]<br>6) GFP (Abcam, ab6556): Rabbit. IHC-P, Electron Microscopy, ICC, IP, Flow Cyt, IHC-Fr, WB. [https://www.abcam.com/gfp-antibody-ab6556.html]<br>7) GPF (Abcam, ab6673): Goat: WB, IP, ELISA, ICC/IF, IHC-P, IHC-FrFl, IHC-Fr. [https://www.abcam.com/gfp-antibody-ab6673.html]<br>8) β-actin (GeneTex, GTX26276): Mouse. WB, ICC/IF, IHC-P, IP, ELISA. [https://www.genetex.com/Product/Detail/beta-Actin-antibody-AC-15/GTX26276]<br>9) β-actin (Cell Signaling Technology, 8H10D10): Mouse. WB, IHC-P, IF-IC, F. [https://www.cellsignal.com/products/primary-antibodies/b-actin-8h10d10-mouse-mab/3700]

Eukaryotic cell lines

Policy information about cell lines

Cell line source(s)	Five cell lines, 4T1, Hepa 1-6, BNL-MEA (BNL 1ME A.7R.1), MCF7 and LS174T, were acquired from ATCC.
Authentication	The mouse cell lines were authenticated with CO1 barcoding and the human cell lines were authenticated with short tandem repeat profiling.
Mycoplasma contamination	Cell lines were confirmed to be negative for mycoplasma contamination using the MycoAlert PLUS kit.
Commonly misidentified lines (See ICLAC register)	No commonly misidentified cell lines were used in the study.

Animals and other organisms

Policy information about studies involving animals; ARRIVE guidelines recommended for reporting animal research

Laboratory animals	Two mouse (Mus musculus) strains were used in the study, BALB/c and C57L/J. Both male and female mice, aged four to seven weeks, were used. Mice were housed under a 12 h light/dark cycle at controlled room temperature of 72 °F and a relative humidity of 60%.
Wild animals	The study did not involve wild animals.
Field-collected samples	The study did not involve samples collected from the field.
Ethics oversight	All animal protocols were approved by the UMass Institutional Animal Care and Use Committee (IACUC). The UMass IACUC is guided by the U.S. Animal Welfare Act and U.S. Public Health Service Policy. UMass Amherst is registered as a research facility (#14-R-0036) with the United States Department of Agriculture and has an approved Animal Welfare Assurance (#A3551-01) on file with the NIH Office of Laboratory Animal Welfare (OLAW).

Note that full information on the approval of the study protocol must also be provided in the manuscript.

Flow Cytometry

Plots

Confirm that:

- [x] The axis labels state the marker and fluorochrome used (e.g. CD4-FITC).
- [x] The axis scales are clearly visible. Include numbers along axes only for bottom left plot of group (a 'group' is an analysis of identical markers).
- [x] All plots are contour plots with outliers or pseudocolor plots.
- [x] A numerical value for number of cells or percentage (with statistics) is provided.

Methodology

Sample preparation	Cells were processed into a single cell suspension with 0.05% trypsin and centrifuged in media for 10 minutes. Cells were fixed in 2% formalin in PBS for 5 minutes. Cells were then permeabilized in 0.1% trypsin and stained with FITC-Salmonella and anti-myc followed by a Dylight 755 secondary antibody against the myc antibody.
Instrument	Dual LSRFortessa, Becton Dickinson
Software	BD FACSDiva

Cell population abundance	Only 4T1 cells from in vitro cell culture were used for flow cytometry experiments and greater than 99% of cells stained positive for the epithelial marker, EPCAM.
Gating strategy	Forward Scatter was first used to gate on cells and exclude debris (P1). Forward scatter-A/Forward scatter-H gating was performed on P1 to isolate single cells (P2). Fluorescence minus one gating was performed on both fluorophores (FITC-Salmonella and Dylight 755) to define the border between the positive and negative populations.

☒ Tick this box to confirm that a figure exemplifying the gating strategy is provided in the Supplementary Information.
