# Supplementary material for: Intracellular delivery of protein drugs with an autonomously lysing bacterial system reduces tumor growth and metastases
Source: Nat Commun. 2021 Oct 21;12:6116. doi: 10.1038/s41467-021-26367-9 (PMC8531320; doi:10.1038/s41467-021-26367-9)
Supplement: Supplementary file 4 — Description of Additional Supplementary Files [file 41467_2021_26367_MOESM4_ESM.pdf]

Title: Supplemental Movie 1.

Description: Time-lapse of GFP release and diffusion into the cytoplasm of cancer cells from ID Salmonella. In early frames, clusters of ID Salmonella are visible inside several MCF7 cancer cells. The bacteria had invaded into the cells prior to the beginning of the clip. The nuclei of the cancer cells are visible as large transparent structures. With time, the ID Salmonella lyse, and the GFP synthesized by the bacteria diffuses throughout the cytoplasm of the cells. The duration between each of the 33 images in the time-lapse is 1 minute.
